# The Fallacy of Univariate Solutions to Complex Systems Problems

**DOI:** 10.3389/fnins.2016.00267

**Published:** 2016-06-08

**Authors:** Christina N. Lessov-Schlaggar, Joshua B. Rubin, Bradley L. Schlaggar

**Affiliations:** ^1^Department of Psychiatry, Washington University School of MedicineSt. Louis, MO, USA; ^2^Department of Neurology, Washington University School of MedicineSt. Louis, MO, USA; ^3^Department of Pediatrics, Washington University School of MedicineSt. Louis, MO, USA; ^4^Department of Radiology, Washington University School of MedicineSt. Louis, MO, USA; ^5^Department of Neuroscience, Washington University School of MedicineSt. Louis, MO, USA

**Keywords:** Tourette syndrome, neuropsychiatric illness, cancer, fMRI, developmental cognitive neuroscience

## Abstract

Complex biological systems, by definition, are composed of multiple components that interact non-linearly. The human brain constitutes, arguably, the most complex biological system known. Yet most investigation of the brain and its function is carried out using assumptions appropriate for simple systems—univariate design and linear statistical approaches. This heuristic must change before we can hope to discover and test interventions to improve the lives of individuals with complex disorders of brain development and function. Indeed, a movement away from simplistic models of biological systems will benefit essentially all domains of biology and medicine. The present brief essay lays the foundation for this argument.

## Introduction

Non-invasive neuroimaging has invigorated a deep and abiding interest in understanding the human brain, the most complex biological system, in health and disease. This burgeoning research focus has impelled technological innovation in neuroimaging and application of a growing number of mathematical/computational approaches to analysis, which help visualize the complexity of the brain in greater depth than previously possible. From our current vantage point we are compelled to ask whether our capabilities have outstripped the paradigms we use for scientific research, and whether our conceptual and analytical frameworks have become a barrier to understanding complex systems.

A deep understanding of complex biological systems requires conceptual and analytical strategies that respect that complexity. Yet, there continues to be a dominating focus in experimental design and analysis on univariate, linear, and narrowly defined relationships. These approaches, including multivariate linear regression (which is an elaboration on the univariate linear framework), are gratifying because they are conceptually simple and align neatly with the traditional scientific method, in which emphasis is placed on a single isolatable dependent variable. However, the univariate/linear approach will necessarily fail when tasked with providing the basis for deep explanations for complex biological systems.

This essay highlights the need to recognize the fallacy of the univariate conceptual framework with respect to complex systems and to embrace complexity so as to align the problem to be solved with the approach taken. We contend that there are some effective ways to study complex systems through care in study design and sample ascertainment, deep phenotyping, and statistical approaches. However, the shift to individual-level analysis, the basis for personalized medicine, will require both methodological advances and a readiness for investigators and reviewers to eschew biologically implausible reductionist models of complex biology.

## Argumentation

### Study design and sample ascertainment

Standard clinical trial design emphasizes a univariate conceptual framework—as the Consolidated Standards of Reporting Trials approach argues (Moher et al., 2001), if randomization is done correctly, the only difference between a treatment and control group is the treatment itself. Then, results are framed to reflect the central tendency of the two groups and whether that central tendency differs for the defined primary outcome. However, the central tendency of a treated group does not necessarily inform the clinician whether the patient currently in the exam room and seeking help is (or is not) likely to respond to the offered therapy, particularly if the patient would not have met study inclusion criteria.

Why would the patient have not been offered entry into the study? Because the study design, inspired by univariate approaches to complex problems, mandates inclusion/exclusion criteria that reduce variability and remove potentially confounding factors, which necessarily makes the study less generalizable to the broader population. Further, it undermines the ability, using study data, to make predictions about treatment response for individual patients. Such single patient/subject level prediction, it seems to us, should be a fundamental and significant priority of clinical trials. Yet, quantifying and characterizing the central tendency at the group level appear to be the principal objectives.

Similarly, a commonly employed study design in cognitive neuroscience is between-group comparison of cases and controls. For some of the authors, case status might comprise tobacco-dependent cigarette smokers or patients with Tourette syndrome (TS), a neurodevelopmental disorder defined by the chronic presence of motor and vocal tics. Controls, by definition, would include non-smokers or individuals without TS, respectively. Comparing cases and controls on brain outcomes would almost certainly uncover group differences (Azizian et al., 2009; Rickards, 2009; Eichele and Plessen, 2013; Fedota and Stein, 2015). However, group differences cannot be ascribed to case/control status alone: both tobacco dependence and TS are complex disorders that do not exist simply on the background of an otherwise typically developed, neuropsychiatrically healthy individual. Tobacco dependent smokers, relative to non-smokers are more likely to abuse other substances (Madden and Heath, 2002; John et al., 2003; Agrawal et al., 2012), to have history of mood or behavioral problems (Grant et al., 2004; Smith et al., 2014), to experience worse socioeconomic indicators, and to have family history of substance use and psychopathology (Lessov et al., 2004; Lawrence et al., 2007; Buu et al., 2009; Xian et al., 2010; CDC, 2011; Zoloto et al., 2012). TS patients, compared to non-TS patients, are more likely to suffer from anxiety and mood disorders, obsessive compulsive disorder (OCD), attention deficit hyperactivity disorder (ADHD), sleep disorders, learning disability, and to have family history of such problems (Mathews and Grados, 2011; O'Rourke et al., 2011; Martino et al., 2013; Mol Debes, 2013; Ghosh et al., 2014; Eysturoy et al., 2015; Hirschtritt et al., 2015). In addition, case status may be but one manifestation that is overt at the moment of investigation. For example, a tobacco dependent adolescent's mood disorder may be subclinical at the time of investigation but emerge later. Or a 2nd grader with a persistent tic disorder may not manifest OCD clinically until middle school. The later emergence of those clinical manifestations belies an earlier determination that the individual is truly free of those clinical burdens. In monogenic genetic disorders, such as Rett Syndrome and CDKL5 epileptic encephalopathy, some individuals with the classic mutation do not necessarily manifest the phenotype (Amir et al., 2000) or may have distinctly different developmental trajectories (Hagebeuk et al., 2015) despite having identical mutations.

Care in sample ascertainment can minimize group differences. One epidemiologically sound approach is to recruit cases and controls from the same demographic area to match socioeconomic characteristics. An alternative approach is to collect sufficient information during screening of potential study participants to identify cases and controls that are matched/similar on background characteristics and to invite the matched subset of participants into the study. One caveat in matching unrelated cases and controls is that individuals who can be matched may represent the tail end of their respective distribution. For example, dependent smokers who can be matched to non-smokers likely do not have burden from known comorbidities and may not be representative of the average dependent smoker; conversely, non-smokers who can be matched to smokers may have greater psychiatric history than the average non-smoker. Another robust approach is to use control individuals who are related to the cases, such as twin or full siblings, to match more closely on genetics, family environment, and other shared history (Lessov-Schlaggar et al., 2013).

In clinical trials, whether random assignment to treatment or control conditions achieves its intended balance is commonly not tested. The commonly employed stringent inclusion criteria that effectively homogenize the study sample likely contribute to the sense of balance in group differences. For example, suppose treatment and control groups are matched on sex (equal numbers of males and females in each group), and socioeconomic status (SES) (equal numbers from low and high SES in each group). On the surface, it would seem that as a consequence of this matching strategy, sex or SES, individually, could not be driving a treatment effect. However, it remains plausible that a sex by SES interaction is lurking such that for the treatment group, 70% of females come from a high SES environment while for the control group, 30% of females come from a high SES environment. Thus, a treatment effect could be driven by a sex by SES interaction that is misattributed. Vigilance in sample ascertainment shows respect for the complexity of human behavior and the neurobiological mechanisms that generate it.

### Deep phenotyping

The co-occurrence of two or more problems is the rule and not the exception in pediatric neuropsychiatric illness (Arcelus and Vostanis, 2003). Comorbidity can be due to shared genetic or environmental mechanisms (Mathews and Grados, 2011; Vrieze et al., 2012), suggesting shared etiology and shared neurobiological mechanisms. For example, brain mechanisms of cognitive control (itself a complex construct) have been implicated in numerous conditions, including drug addiction and TS (Kalivas and Volkow, 2005; Mueller et al., 2006; Church et al., 2009; Garavan and Weierstall, 2012; Jung et al., 2013). Therefore, when comparing cases and controls care in the kind and amount of phenotypic data collection is also necessary. Having data on risk factors allows not only for better matching algorithms, but also for exploration of phenotypic subgroups that differ in behavioral phenomenology. For example, using multiple measures in a large family study of TS, latent class analysis identified five TS subgroups characterized by TS+OCD+ADHD, TS+OCD, TS plus obsessive compulsive behaviors, chronic tics plus OCD, and a subgroup with minimal symptomatology (Grados and Mathews, 2008). Further, only the TS+OCD+ADHD subgroup was significantly heritable (Grados and Mathews, 2008). The differential clustering of symptoms, diagnoses, and heritability estimates, suggest differences in disease etiology or similar proximate etiological mechanisms but disparate additional modifying factors. Identifying potential differences in etiology and modifying factors is paramount to the task of identifying effective therapy. If there is an assumption that all TS manifests from the same underlying cause, then it would necessarily follow—down a garden path argument—that all patients with TS should respond to the same therapy. Of course, inter-individual differences in response to therapy are obvious; such differences could be the consequence of TS as a phenocopy for different etiologies, or could be the consequence of genetic polymorphisms in drug metabolism pathways, unrelated to the etiology of TS. Approaches to understanding therapy optimization require reorienting our approach to investigation so as to determine the reasons that a given patient responds to treatment B and not treatment A.

It is important to recognize that heterogeneity is not limited to atypical populations. It may be discomforting to realize that the composition of a standard group of “healthy controls” is almost certainly heterogeneous. For example, Fair et al. (2012) applied a large neuropsychological battery to a cohort of typically developing children collected as a control sample in a study of ADHD. They then applied an unsupervised clustering algorithm to the psychometric data of each individual and identified subgroups within the cohort of healthy controls that mirrored the subgrouping identified for the ADHD cohort (Fair et al., 2012). The implications of clustering individuals into subgroups based on rich single subject data are substantial given that the standard case/control statistical analysis assumes (incorrectly, most likely) that the case and control groups are each representative of the population of cases and controls, allowing for the application of standard parametric statistics to test group differences.

In another example, using resting state functional connectivity MRI data, groups of typically developing children and children with ADHD could be separated into subgroups based on the pattern of functional connectivity of the nucleus accumbens with the rest of the brain (Costa Dias et al., 2015). Differences between controls and ADHD patients within each subgroup showed different aspects of atypical connectivity in ADHD (Costa Dias et al., 2015); the ADHD subgroup demonstrating atypical connectivity of the nucleus accumbens with attention networks also had higher impulsivity relative to respective controls and to the other ADHD subgroups (Costa Dias et al., 2015) suggesting distinct mechanism(s) that may underlie impulsivity in ADHD.

Using resting state functional connectivity MRI, Laumann and colleagues showed that collecting data from the same individual over multiple occasions achieves high level of measurement accuracy and uncovers individual-specific functional brain organization (Laumann et al., 2015). The functional organization of the individual brain shares similarity to group-level functional organization, in that functional systems are evident on the individual and group-average brain (Laumann et al., 2015). However, the functional organization of the individual brain shows a more complex landscape where adjacent cortical regions belong to two or more functional systems, and not one system as in the group-average brain, as well as differences in functional system boundaries between right and left hemispheres (Laumann et al., 2015). This level of specificity could only can be achieved using a large amount of data from the same individual (see also Poldrack et al., 2015) showing how such an approach can detect inter-individual differences that might be associated with individual differences in behavior, disease mechanism, treatment response, and so forth.

Analysis that embraces complexity provides a richer, more interesting, and likely more biologically relevant model of causal mechanisms. By liberalizing phenotypic definitions and collecting as much data per individual as possible, we will be able to better understand individual differences and to better identify deviant or rare phenotypes.

### Statistical approaches

Often, we see lack of capitalizing on good study design or deep phenotyping when it comes to statistical analysis, such as longitudinal data being analyzed cross-sectionally or comorbidity being treated as a confounding variable. Treating longitudinal data as cross-sectional does not take advantage of the overall reduction in variability and error estimation with repeat assessment of the same individuals. A small mean difference in task-evoked brain activity, as measured by fMRI, between times 1 and 2 may not be significant when analyzed cross-sectionally; however, if each subject's low amplitude response moved in the same direction, the effect could be highly statistically significant when analyzed longitudinally.

Comorbidity is often treated as nuisance variable(s). Investigations by the Tourette Syndrome Association International Consortium for Genetics demonstrate that the neuropsychiatric comorbidities of TS have very complex genetic relationships (Mathews and Grados, 2011). It is simply erroneous to consider comorbidities to superimpose linearly on the diagnosis of interest. Yet the practice of using linear regression or covariance to remove the confounding contribution of a comorbid diagnosis is predicated on such a linear relationship. Comorbidity shares variation with the phenotype of interest that affects outcome, and treating it as a nuisance variable undermines the results by statistically removing informative variation. Further, a covariate only controls for the linear relationship of that variable with outcome. It is likely the case that comorbidity is not captured by additive effects, but is the result of complex interactions of etiological mechanisms. The notion of pure insertion of a phenotype, such as OCD, onto TS, is problematic. In the neuroimaging literature, Friston et al. (1996) discussed the problem of pure insertion in the setting of cognitive subtraction, refuting the implicit assumption employed in many neuroimaging studies that “there are no interactions among the cognitive components of a task.”

In the clinical setting, often the most vexing question asked by the parent of a child diagnosed with a persistent tic disorder is “what does the future hold for my child?” The ability to provide real, evidence-based, predictions for that patient and family—not a summary relevant to the central tendency of the population of individuals with persistent tics, but predictions that are specific to the patient in the office—is of paramount importance. Single patient/subject level prediction requires methodological approaches to study design and data analysis that capitalize on the richness afforded by high dimensional data and inter-individual variance, as shown in Laumann et al. (2015), for example.

Our own first efforts in this regard used resting state functional connectivity MRI and support vector machine based multivariate pattern analysis to predict, on a single subject basis, age-group membership (adult vs. child) as well as the brain maturity of single subjects (Dosenbach et al., 2010; Greene et al., 2014). We have applied similar approaches to predict whether an individual has TS or not (Greene et al., 2016). These approaches (Johnston et al., 2015; Kambeitz-Ilankovic et al., 2015; Stock et al., 2015) are orienting the field toward the importance of single subject/patient level prediction. Fair and colleagues (Miranda-Dominguez et al., 2014) introduced a highly compelling recent exemplar, “connectotyping,” using resting state functional connectivity MRI, to reveal a functional “fingerprint” of an individual with substantially less data than needed for the deep characterization described in Laumann's (Laumann et al., 2015) and Poldrack's work (Poldrack et al., 2015).

One important caveat regarding multivariate pattern analysis is that, at least to our knowledge, it is not possible to perform what would be considered a standard power analysis—constructs like effect size and measurement variance do not readily translate into the n-dimensional space within which such analysis operates.

### Beyond the cognitive neurosciences

Potential advantages of single subject, rather than group-level prediction, are important to consider in other diseases with complex phenotypes, like cancer. Driving forces for clinical trial design and conduct included, ethical considerations, statistical models and simplicity in order to ensure consistency across multiple trial sites (Meier, 1975). Missing from the driving forces for trial design is disease biology. Advances in cancer biology have significantly refined our view of causation, such that histological diagnoses are giving way to molecular subtyping within histological diagnostic groups, and patients are being stratified for therapies that target causative genetic events (Bautista et al., 2014; Robinson et al., 2015). While this approach is touted as the foundation for personalized “precision” medicine, in reality it most frequently perpetuates a monolithic view of cancer biology and therapeutic responses that is inconsistent with the state of scientific evidence in cancer biology.

As we are focused here on central nervous system disease we will limit our comments to malignant brain tumors. The combination of surgery, radiation and chemotherapy for glioblastoma, the most common and malignant of brain tumors, was first applied in the late 1940s and first studied in clinical trials in the 1960s (Gunther, 1949; Levin and Wilson, 1976; Walker and Gehan, 1976). The improvement in survival was measured in months and for the vast majority of patients this remains the benefit of therapy today (Stupp et al., 2005). Over the same period of time, our understanding of the biology of glioblastoma has advanced remarkably. The complexity of the mutational landscape has been repeatedly described (Frattini et al., 2013; van Thuijl et al., 2015). The significance of epigenetic modulation of the cancer genome to cancer biology and therapeutic resistance has been recognized (Sturm et al., 2014). The importance of multi-clonality to tumor evolution in response to treatment has been established (Kim et al., 2015), as has impact of cancer immune editing and immunological checkpoints to cancer development (Pellegatta et al., 2011). We also know that this spectrum of intra-tumoral heterogeneity in each patient must also be overlaid on the distinct biologies of males vs. females (Sun et al., 2014) and genome-wide polymorphisms that determine important phenotypic differences between individuals in such things as metabolism and circadian rhythm, which impact on disease risk, progression, and therapeutic responses.

Among the conclusions of this enormous body of research is that each glioblastoma patient has multiple genetically and epigenetically distinct clonal lineages that must be simultaneously targeted for a reasonable chance of cure. Despite this knowledge, we continue to “match” groups of patients and evaluate novel drugs one at a time, and we continue to dramatically fail to improve outcome (Bastien et al., 2015). We have neglected to recognize that the complexity of this disease demands a revolutionary change in approaches to clinical investigation in which the individual is what is being interrogated, not the group. Success may require abandoning current research paradigms and statistical frameworks in favor of models that can be informative for multiple “n's” of one.

## Concluding remarks

Classical statistics, developed before computers and technologies that can analyze and deliver millions of data points, may be inadequate for analyzing high-dimensional data sets. Inherent in the idea of personalized medicine is a translational approach, whereby basic science and clinical research data can be used together to predict with high accuracy an individual patient's clinical prognosis and treatment. Achieving personalized medicine will almost certainly require a paradigm shift toward embracing complexity and developing and funding complex systems analytics research.

## Author contributions

BS co-conceived the thesis of this essay and co-authored it. CL co-conceived the thesis of this essay and co-authored it. JR co-conceived the thesis of this essay and co-authored it.

## Funding

This effort was supported by NIH K01DA027046 (CL), R01CA136573 (JR), R01HD057076 (BS), and the Intellectual and Developmental Disabilities Research Center at Washington University U54HD087011 (CL, BS).

### Conflict of interest statement

The authors declare that the research was conducted in the absence of any commercial or financial relationships that could be construed as a potential conflict of interest.
